# High Genetic Diversity despite Conserved Karyotype Organization in the Giant Trahiras from Genus *Hoplias* (Characiformes, Erythrinidae)

**DOI:** 10.3390/genes12020252

**Published:** 2021-02-10

**Authors:** Francisco de M. C. Sassi, Manolo F. Perez, Vanessa Cristina S. Oliveira, Geize A. Deon, Fernando H. S. de Souza, Pedro H. N. Ferreira, Ezequiel A. de Oliveira, Terumi Hatanaka, Thomas Liehr, Luiz A. C. Bertollo, Marcelo de B. Cioffi

**Affiliations:** 1 Laboratório de Citogenética de Peixes, Departamento de Genética e Evolução, Universidade Federal de São Carlos, 13565-905 São Carlos, SP, Brazil; francisco.sassi@hotmail.com (F.d.M.C.S.); manolofperez@gmail.com (M.F.P.); vanessacristina.sales@gmail.com (V.C.S.O.); geizedeon@hotmail.com (G.A.D.); fernando_Hsouza@outlook.com.br (F.H.S.d.S.); pferreira@estudante.ufscar.br (P.H.N.F.); hterumi@yahoo.com.br (T.H.); bertollo@ufscar.br (L.A.C.B.); mbcioffi@ufscar.br (M.d.B.C.); 2 Secretaria de Estado de Educação de Mato Grosso—SEDUC-MT, 78049-909 Cuiabá, MT, Brazil; ezekbio@gmail.com; 3 Institute of Human Genetics, University Hospital Jena, 07747 Jena, Germany

**Keywords:** fishes, cytogenetics, DArTseq, phylogenetics, genomics

## Abstract

In the fish genus *Hoplias*, two major general groups can be found, one of which is formed by the “common trahiras” (*Hoplias malabaricus* group) and the other by the “giant trahiras” (*Hoplias lacerdae* group, in addition to *Hoplias aimara*), which usually comprises specimens of larger body size. Previous investigations from the giant trahiras group recovered 2n = 50 meta/submetacentric chromosomes and no sex chromosome differentiation, indicating a probable conservative pattern for their karyotype organization. Here, we conducted comparative cytogenetic studies in six giant trahiras species, two of them for the first time. We employed standard and advanced molecular cytogenetics procedures, including comparative genomic hybridization (CGH), as well as genomic assessments of diversity levels and phylogenetic relationships among them. The results strongly suggest that the giant trahiras have a particular and differentiated evolutionary pathway inside the *Hoplias* genus. While these species share the same 2n and karyotypes, their congeneric species of the *H. malabaricus* group show a notable chromosomal diversity in number, morphology, and sex chromosome systems. However, at the same time, significant changes were characterized at their inner chromosomal level, as well as in their genetic diversity, highlighting their current relationships resulting from different evolutionary histories.

## 1. Introduction

The neotropical freshwater ichthyofauna encompasses more than 5200 species distributed from the southern United States to southern Argentina, in which the order Characiformes, with almost 2300 species, stands out as a notable representative of this biodiversity [1,2,3]. Erythrinidae is a characiform family with few species, but displays a large geographic distribution throughout South America [4]. Broad distributions, associated with gene flow restrictions, generally offer suitable conditions for the fixation of distinct evolutionary characteristics among distant populations. This condition is even more remarkable for freshwater fishes since their dispersal can be limited and often confined to isolated hydrographic systems [5]. Such a situation is particularly true for the Erythrinidae family, in which intra-specific chromosomal differentiation has been highlighted for several of its representatives [6,7]. A significant amount of cytogenetic data highlight the diversity displayed by populations of its three genera, including karyotypes with different diploid numbers (2n), chromosomal morphology, and sex chromosome systems, some of which are already well differentiated while others display an early stage of differentiation [6,7,8,9,10,11,12].

Despite such remarkable cytogenetic diversity, only 18 species are currently recognized for this family, distributed in three genera: *Erythrinus* (2), *Hoplerythrinus* (3), and *Hoplias* (13) [4,13,14]. This likely does not reflect the real taxonomic status of the Erythrinidae family, if we consider the chromosomal diversity observed within several of its species (revised in [15]). Particularly, two major general groups can be found in the genus *Hoplias*, the “common trahiras” (*Hoplias malabaricus* group), and the “giant trahiras” (*Hoplias lacerdae* group; [13]). For the first group, a considerable amount of cytogenetic data highlight the meaningful intra-specific diversity that occurs in the *H. malabaricus* nominal species. Indeed, seven major karyomorphs (i.e., exclusive and different karyotypes) have been identified until now [7,8,10,12,16], indicating a probable species complex and the need for a taxonomic review of this group.

Conversely, concerning the giant trahiras, five species are currently recognized in the *Hoplias lacerdae* group based on their differential meristic and morphological aspects, namely *Hoplias australis*, *Hoplias brasiliensis*, *Hoplias curupira*, *Hoplias intermedius,* and *H. lacerdae* [13]. This group presents the medial margins of the dentaries showing a somewhat parallel position and the absence of teeth in the basihyal, unlike what occurs in the *H. malabaricus* group [13]. Notably, and distinct to what is found in the *H. malabaricus* group, *H. lacerdae* species are characterized by a more stable karyotypic organization, both at the numerical and structural levels [17,18]. In the most recent assessment, three out of its five species (*H. lacerdae*, *H. brasiliensis,* and *H. intermedius*) were investigated [19]. All evaluated species showed 2n = 50 meta- and submetacentric chromosomes, with undifferentiated sex chromosome systems. Besides, there were similar distribution patterns for the C-positive heterochromatin and some interspecific differentiations were evidenced from the chromosomal mapping of repetitive DNA sequences [19]. Interestingly, another large trahira species, *Hoplias aimara*, for which the taxonomy has already been revised and validated [20], but not inserted into the *H. lacerdae* group due to its morphological features [13], also shares these karyotypic features [19,21].

In the present study, we conducted comparative cytogenetic studies in six giant trahira species, three of them examined for the first time, using conventional and advanced molecular cytogenetic methods. Then, we compared our cytogenetic results to those recovered with genotyping by high-throughput sequencing technology, to explore diversity at the chromosomal and genomic level. The resulting data allowed us to characterize their chromosomal evolutionary trends, highlighting their genomic relationships, and uncovering chromosome homologies and differences among them.

## 2. Materials and Methods

### 2.1. Individuals, Collection Sites, and Classical Cytogenetic Methods

**Figure 1:**
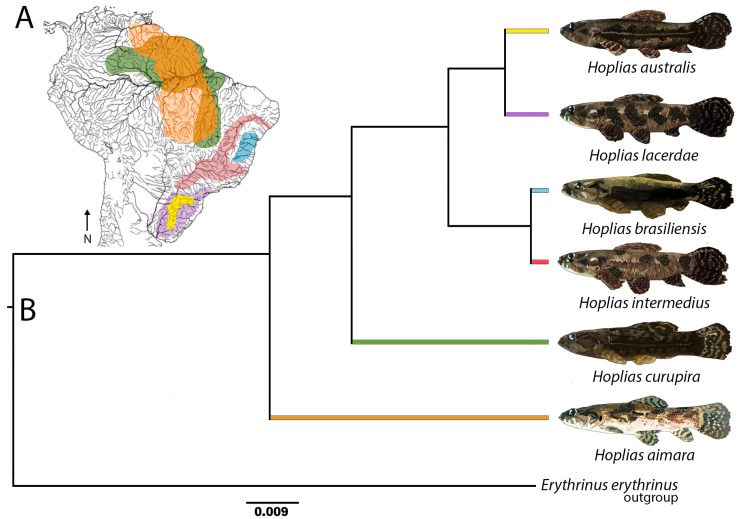
Phylogenetic relationships of giant trahiras and their distribution in South America. (**A**)—Distribution map of giant *Hoplias* species (*H. lacerdae* group + *H. aimara*), according to data from [20] and [13]. Each color represents one species, as follows: yellow, *Hoplias australis*; purple, *H. lacerdae*; blue, *H. brasiliensis*; red, *H. intermedius*; green, *H. curupira*; orange, *H. aimara*. (**B**)—Species tree recovered with SNAPP [22], based on DArTseq data, using an *Erythtrinus erythrinus* specimen as outgroup. The color scheme at the tree terminal branches follows the same used in the map. Fish species were illustrated by Vitor Augusto Rezende dos Santos using Adobe Photoshop CC 2020 and Adobe Illustrator CC 2020.

We analyzed five species of the *H. lacerdae* group, namely *Hoplias lacerdae*, *H. brasiliensis*, *H. intermedius*, *H. curupira,* and *H. australis*, the former two for the first time. Another giant trahira, *H. aimara*, was also re-investigated. Figure 1A and Table 1 depict the Brazilian distribution of species and the number of individuals studied.

Mitotic chromosomes were obtained by the air-drying protocol [23] using kidney cells after in vivo colchicine treatment. The detection of C-positive heterochromatin (C-banding) followed [24] and the staining of Ag-NOR bands, [25]. In the latter, the formic acid plus gelatin solution was applied twice, and the glass slide was placed on a heating plate at 65 °C for about 3.50 min, or until the chromosomal spreads turned into a brown/caramel aspect.

### 2.2. Fish-Based Experiments

Probes for fluorescence in situ hybridization (FISH) were obtained from nuclear DNA previously cloned into plasmid vectors and propagated in competent cells of *Escherichia coli* DH5α (Invitrogen, San Diego, CA, USA). The 5S rDNA probe included 120 base pairs (bp) of the RNAr 5S codificant gene and 200 bp of a non-transcribed spacer (NTS), isolated according to [26]. The 18S rDNA probe contained a 1400 bp segment of the rRNA 18S gene, isolated according to [27]. Both probes were directly labeled with the Nick-Translation mix kit (Jena Bioscience, Jena, Germany). The 5S rDNA probe was labeled with Atto550-dUTP and the 18S rDNA one with AF488-dUTP, according to the manufacturer’s manual. Small repetitive sequences ((A)_30_, (CA)_15_, (GA)_15_, and (CAC)_10_) were directly labeled with Cy-3 during the synthesis, as described by [28]. The FISH experiments followed high stringency conditions, as related in [29].

Comparative genomic hybridization (CGH) experiments were performed using genomic DNA of all species analyzed on metaphase chromosomes of *Hoplias intermedius* as a reference. The female-derived gDNA of all species was extracted from liver tissue following [30]. In all assays, the gDNA of *H. intermedius* was directly labeled with AF488-dUTP (green fluorescence) using a Nick-translation-based labeling kit (Jena Bioscence, Jena, Germany), and the gDNA of *H. aimara*, *H. australis*, *H. brasiliensis*, *H. lacerdae,* and *H. curupira* was directly labeled with Atto550-dUTP (red fluorescence), also using the nick-translation labeling kit. The final hybridization mix contained 500 ng of *H. intermedius* gDNA plus 500 ng of gDNA from the other species. Blocking of repetitive sequences was achieved using 15 μg of C0t-1 female-derived DNA from each species [31] dissolved in 20 μL of hybridization buffer containing 50% formamide plus 2× saline-sodium citrate (SSC), in addition to 10% sodium dodecyl sulphate (SDS), 10% dextran sulphate, and Denhardt’s buffer (pH 7.0). The ratio of probe vs. C0t-1 was based on several other CGH experiments conducted in fish groups [16,32,33,34,35].

The 2n value, karyotype structure, and CGH results were confirmed by the analysis of at least 30 metaphase spreads per individual. Images were captured using an Olympus BX50 microscope (Olympus Corporation, Ishikawa, Japan), with a CoolSNAP camera and processed using Image-Pro Plus 4.1 software (Media Cybernetics, Silver Spring, MD, USA). Chromosomes were classified as metacentric (m), submetacentric (sm), subtelocentric (st), or acrocentric (a), according to [36], and were organized in the figures using Adobe Photoshop CC 2020.

### 2.3. DArTseq Genotyping and Genetic Diversity

To perform DNA sequencing, liver fragments from all samples presented in Table 2 were sent to Diversity Arrays Technology Pty Ltd. (Canberra, Australia). The DArTseq procedure consists of a complexity reduction method based on the use of a frequently cut enzyme (*Sph*I), and a methylation-sensitive enzyme (*Pst*I). Prepared libraries were then sequenced on an Illumina HiSeq2500 platform. Raw sequence data were analyzed in ipyrad v.0.9.59 for quality filtering and recovery of SNP (Single Nucleotide Polymorphism) genotypes. Specifically, sequences with less than 35 base pairs or with more than four low-quality bases (considering Q < 20) were discarded and sequencing adapters were trimmed. Then, the pipeline performed a de novo clustering and aligned the reads for each sample separately, retaining only clusters with six or more reads as a pre-locus. The obtained pre-loci for each sample were then clustered between all samples. Clusters present in all samples were classified as definitive loci and only unlinked markers were retained by selecting only the SNPs with less missing data from each locus. The resulting SNP matrix was exported and used in further analyses.

Genetic diversity within populations of each species was assessed using the R package dartR [37] by estimating allelic richness (*A*_R_), private alleles (*P*_A_), observed heterozygosity (*H*_O_), expected heterozygosity (*H*_E_), and the Shannon diversity index (*S*—according to [38]).

### 2.4. Genetic Relationships and Phylogenetic Estimation

We also used dartR to visualize the genetic diversity distribution among species with a principal coordinate analysis (PCoA). The phylogenetic relationships were estimated from the SNP matrix by inferring a species tree with the Bayesian software SNAPP v.1.5.1. [39], part of the BEAST v.2.6.3 package [40]. We performed two independent Markov chain Monte Carlo (MCMC) runs of 1 million generations, sampling every 500 generations, with settings and prior values following suggestions from [22]. Specifically, both relative mutation rates were not sampled and assumed to be 1, while the coalescent rate was set with an initial value of 200, and the distributions for populations theta values and lambda were left at the default values. Tracer v1.7.1 [41] was used to check convergence, and a maximum clade credibility tree based on common ancestor heights was generated after a 25% burn-in in TreeAnnotator. FigTree v1.4.3 was used to generate the consensus tree figure.

## 3. Results

### 3.1. Karyotypes, C-Banding, and Chromosomal Mapping of Repetitive Sequences

**Figure 2:**
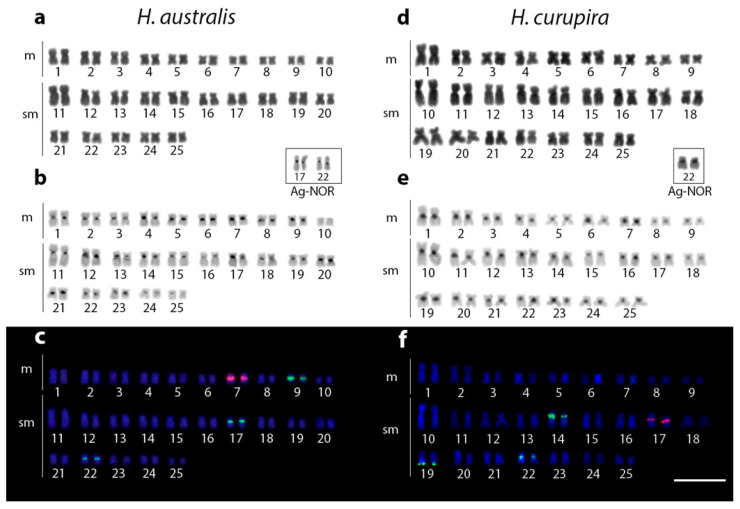
Karyotypes of *Hoplias australis* (**a**–**c**) and *H. curupira* (**d**–**f**) organized from Giemsa-stained (**a**,**d**) and C-banded (**b**),(**e**) chromosomes. Dual-color fluorescence in situ hybridization (FISH) karyotypes with 5S (red) and 18S (green) rDNA probes are also shown for both species (**c**) and (**f**), respectively, with chromosomes counterstained with DAPI (blue). Scale bar = 5 μm.

Both *H. australis* and *H. curupira*, which were not previously cytogenetically assessed, have 2n = 50 metacentric/submetacentric chromosomes, but slightly differing in configuration, with karyotypes composed by 20 m + 30 sm and 18 m + 32 sm, respectively (Figure 2a,d). The C-positive heterochromatin also follows the same distribution pattern for both species, located in the pericentromeric region of all chromosomes (Figure 2b,e).

The mapping of ribosomal regions by FISH highlighted the same number of rDNA sites for both species, although distributed in different chromosomes in the karyotypes. Accordingly, *H. australis* and *H. curupira* present a single chromosome pair with pericentromeric 5S rDNA sequences, the 7th and the 17th ones, respectively. Similarly, the two species also present three chromosome pairs bearing 18S rDNA sequences, all of which are pericentromeric in *H. australis* (pairs 9, 17, and 22). In turn, in *H. curupira*, only two of these chromosomes contain sites with a pericentromeric location (pairs 14 and 22), while the other one has it positioned in the ends of the long arms (pair 19). However, only the 18S sequences located in the chromosomes 17 and 22 of *H. australis* and 22 of *H. curupira* matched with the argyrophilic nucleolar-organizing region (AgNOR) sites, thus indicating that they were the only ones with transcriptional activity in the preceding mitotic cycle (Figure 2c,f).

**Figure 3:**
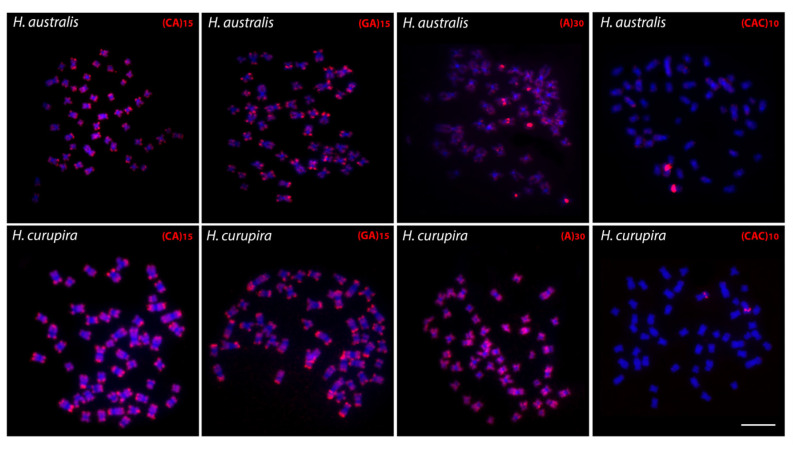
Microsatellite sequences (red) mapped against chromosomes of *Hoplias australis* and *H. curupira*. Chromosomes were counterstained with DAPI (blue). Scale bar = 5 μm.

Both similar and divergent distribution patterns were also detected for microsatellite sequences between *Hoplias australis* and *H. curupira* (Figure 3). For (CA)_15_ and (GA)_15_ probes, both species present a strong hybridization pattern in the telomeric regions of all chromosomes, in addition to some other small pericentromeric and interstitial signals. However, probe (A)_30_ reveals a strong accumulation in the pericentromeric regions of some *H. australis* chromosomes, with the others showing weaker signals in their telomeric regions, in contrast to what occurred in *H. curupira* where the chromosomes are strongly marked in all extension, except in the pericentromeric regions. For the triplet (CAC)_10_, only a single chromosome pair was hybridized in both species, which coincides with the one carrying the 5S rDNA motif (i.e., pair 7 and 17 of *H. australis* and *H. curupira*, respectively).

### 3.2. Comparative Genomic Hybridization (CGH)

**Figure 4:**
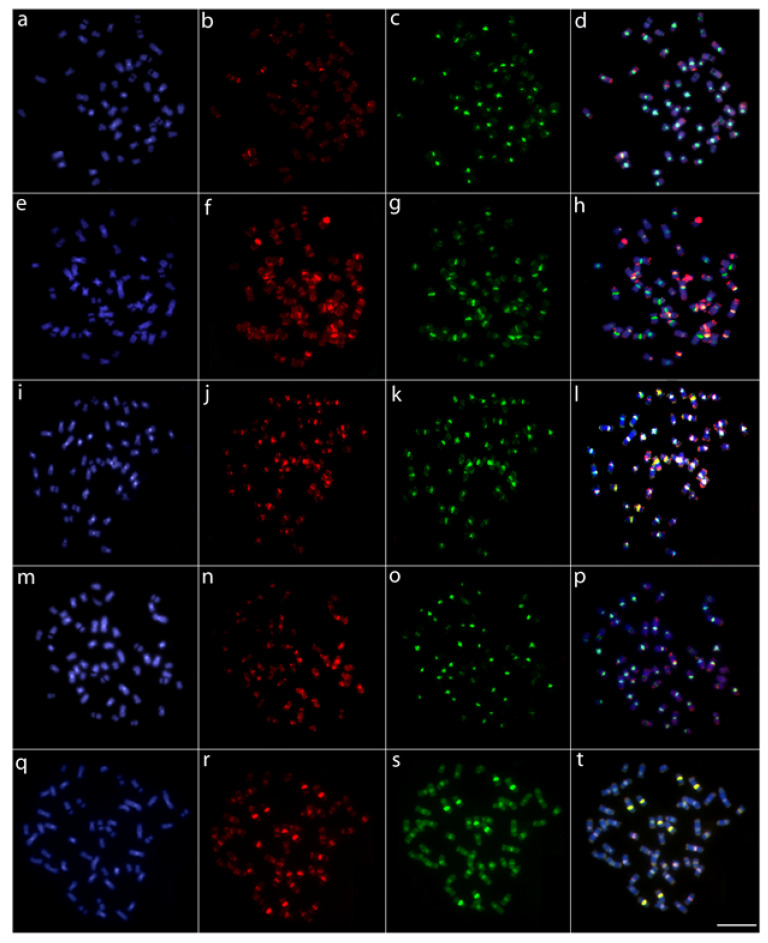
Comparative genomic hybridization using the gDNA of the five *Hoplias* species against the chromosomal background of *H. intermedius*. The first column (**a**,**e**,**i**,**m**,**q**): DAPI images (blue); second column (**b**,**f**,**j**,**n**,**r**): hybridization patterns using gDNA probe from *H. intermedius*; third column (**c**,**g**,**k**,**o**,**s**): hybridization patterns using gDNA probes from *H. aimara*, *H. australis*, *H. brasiliensis*, *H. curupira*, and *H. lacerdae*, respectively; fourth column (**d**,**h**,**l**,**p**,**t**): merged images of both genomic probes and DAPI staining depicting the shared regions in yellow. Scale bar = 5 μm.

The comparative genomic hybridization (CGH) experiments among the six giant trahiras showed that they exhibit divergent genomic patterns, with a high level of compartmentalization and the composition of repetitive sequences varying both in quantity and distribution. However, it was also evidenced that *Hoplias australis* and, especially, *H. brasiliensis*, present a greater number of shared regions with *H. intermedius* than the other three species (Figure 4).

### 3.3. Comparative Analyses Using Diversity Arrays Technology Sequencing Data

**Figure 5:**
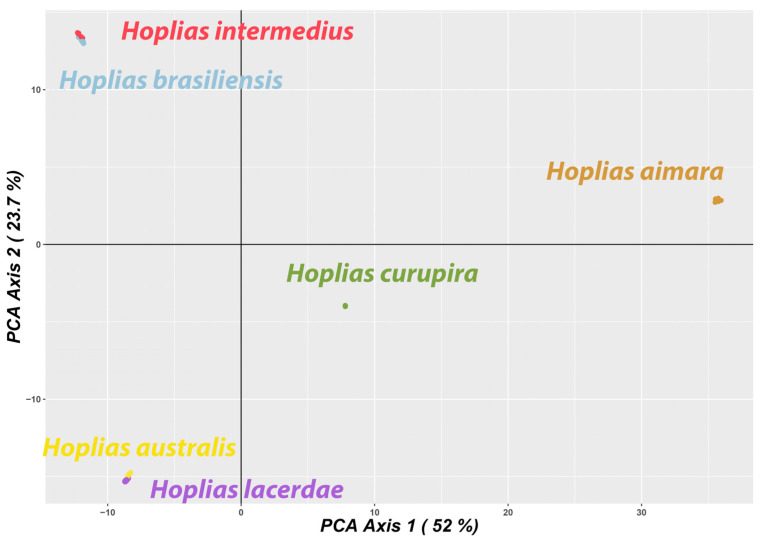
Principal coordinate analysis of giant trahiras. The color scheme for each species follows the same used in Figure 1.

The DArTseq procedure generated approximately 2 million reads per sample. After filtering, the final dataset was composed of 1858 SNPs. Genetic diversity indexes indicated a high differentiation of *Hoplias aimara* and *H. curupira*, as they present a higher number of private alleles compared with the other giant trahiras. In turn, these two species also have the lowest diversity levels, as evidenced by the allelic richness, the heterozygosity (*H*_O_ and *H*_E_), and the Shannon diversity, while *H. australis* and *H. brasiliensis* have the highest ones (Table 2). The PCoA summarized 52.0% of the total variation in the X-axis and 23.7% in the Y-axis. The results indicate that *H. intermedius* and *H. brasiliensis* are more closely related to each other, as are *H. australis* and *H. lacerdae*. In turn, *H. curupira* stands out as the most genetically distant species within the *H. lacerdae* group, while *H. aimara* was the most distant overall (Figure 5). The species tree generated in SNAPP showed this same relationship between species, with posterior probabilities equal to 1 in all nodes. (Figure 1B).

## 4. Discussion

Previous studies indicated that trahiras forming the *Hoplias lacerdae* group share a similar macro-karyotype pattern, with some differentiation among them concerning chromosome types and molecular cytogenetic markers. While *H. lacerdae* has 2n = 50 chromosomes (16m + 34sm), *H. intermedius* and *H. brasiliensis* have 2n = 50 (20m + 30sm) [19]. Here, for the first time, the two other remaining species recognized for this group, namely *H. australis* and *H. curupira*, were cytogenetically analyzed. Both also present 2n = 50 chromosomes, with slight differentiation between them in the karyotype composition, i.e., *H. australis* has 2n = 50 chromosomes (20m + 30sm) and *H. curupira* 2n = 50 (18m + 32sm). Thus, the evolutionary chromosomal trend in the *H. lacerdae* group remains retained (i.e., all species with 2n = 50 meta/submetacentric chromosomes). As the 2n number does not differ among species but only the chromosomal morphology, it is likely that rearrangements that modify the centromere position, such as pericentric inversions or centromere reposition, are the main ones that have driven their chromosomal evolution. Such events can increase the linkage disequilibrium and, when adaptive alleles are included in the inverted regions, the fixation of the rearrangements in the population can be favored [42]. The preferential distribution of the C-positive heterochromatin in the pericentromeric regions, as observed in both *H. australis* and *H. curupira*, is also found in all other giant trahiras [19].

**Figure 6:**
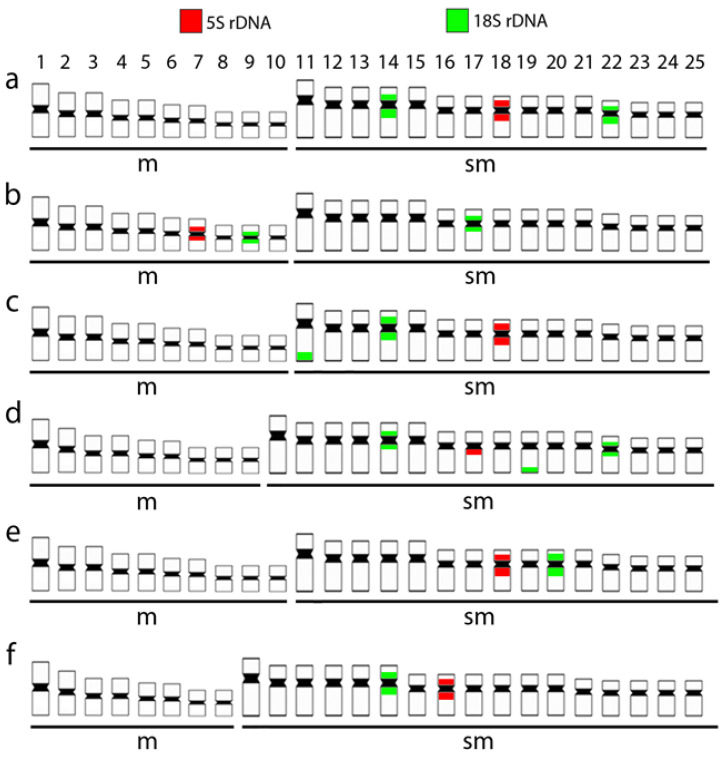
Representative idiograms of (**a**) *Hoplias aimara*; (**b**) *H. australis;* (**c**) *H. brasiliensis*; (**d**) *H. curupira*; (**e**) *H. intermedius*; and (**f**) *H. lacerdae* highlighting the distribution of 18S (green) and 5S (red) rDNA sequences on the chromosomes.

Species’ lifestyle can be a good indicator for understanding karyotype evolution. For the *Hoplias* genus, the largest chromosomal variation occurs in the *H. malabaricus* group that inhabits more lentic environments, while species of the *H. lacerdae* group prefer main river channels as habitat [21]. It is likely that such a condition, contrary to what occurs in lentic environments without large migratory events, may contribute to the maintenance of homogeneous karyotypes, without major macro-structural differences. Several migratory fish families usually show conserved karyotypes concerning the 2n number and chromosomal macrostructure [19,43,44]. Despite this, microstructural and genetic changes may act as significant evolutionary factors, creating probable post-zygotic barriers as documented, for example, in *Centropomus* species [45]. Instead, the conservative chromosomal macrostructure of the *H. lacerdae* group is not maintained in the level of internal chromosomal characteristics. This is evidenced by the differential distributions of the ribosomal DNA sites among species (Figure 6) and the repetitive DNA fraction, as indicated by their comparative genomic hybridization (CGH) analysis. Since repetitive DNA constitutes a highly dynamic fraction of the genome, it has active participation in the evolutionary process leading to significant differentiation, even among closely related species [46,47,48,49,50]. Such differences in the chromosomal mapping of repetitive sequences among the analyzed species were also observed at their genomic levels, with the detection of a high number of private alleles (Table 1), along with high support in all nodes of the species tree (Figure 1A).

The phylogeny reconstructed by [14] points to two groups of species in the *H. lacerdae* group, one of them composed of the northern Brazilian species and the other one containing south/southeast Brazil species. This division can be related to several geological events that shaped the distribution of the Brazilian river basins, such as the extinction of the Michicola Arch in Paleogene and the uplift of the Serra do Mar and mountains in central Brazil, supporting the separation of the northern basins from those of the southern region [51,52,53,54]. The proposition of [14] is also corroborated by our CGH, genomic, and phylogenetic results, since the genome of the southeastern species (*H. intermedius*) shares a greater number of repetitive DNAs with those of the northeastern (*H. brasiliensis*) and south (*H. australis*) species than with that of the north (*H. curupira*) species, as also with *H. aimara*. Accordingly, the comparative genetic diversity also highlights a higher level of differentiation for both *H. curupira* and *H. aimara*, which show the greatest number of private alleles and appear as a sister group of the clade containing all other species. Besides, our genetic data indicate that *H. intermedius* and *H. brasiliensis* are more closely related to each other in the same way that *H. australis* and *H. lacerdae* are. In turn, *H. curupira*, the northernmost species, is the most differentiated within the *H. lacerdae* group, with *H. aimara* being placed as a sister species of the entire *H. lacerdae* group.

Finally, some additional considerations deserve to be addressed concerning *Hoplias aimara*. Three groups of species were recognized by [55] in the genus *Hoplias*: the *H. lacerdae* group (revised by [13]), the *H. malabaricus* group (not yet revised), and the *H. macrophthalmus* group, which was revised by [20] and characterized as monotypic. It was found that *H. aimara* and *H. macrophthalmus* refer to the same taxon, with *H. aimara* being the priority in the denomination according to the Code of Zoological Nomenclature. This species is characterized by missing the accessory ectopterygoid bone, as well as by the occurrence of a characteristic dark oval spot on the opercular membrane [13,20]. Lastly, the data of [14] with DNA barcoding support the indication of *H. aimara* as also belonging to the *H. lacerdae* clade. In this respect, authors consider that “the diagnostic morphological characters of *H. aimara* would only apply at the species level” [14]. Chromosomal data show that *H. aimara* has the same karyotype macrostructure, 2n = 50 meta-submetacentric chromosomes, like all other species of the *H. lacerdae* group [19,21,present study], thus evidencing its closer relationship with the *H. lacerdae* group than with the *H. malabaricus* group. However, despite such proximity, our genetic data indicate that *H. aimara* is a sister clade to all species of the *H. lacerdae* group, highlighting that the conservative 2n = 50 m/sm chromosomes are shared characteristics that are being maintained for a long time in the evolutionary process of the genus *Hoplias*.

## 5. Conclusions

Chromosomal data of the giant trahiras (*Hoplias australis*, *H. aimara*, *H. brasiliensis*, *H. curupira*, *H. intermedius*, *H. lacerdae*) support the conservative status of their diploid number allied to a notable diversity in a genomic scale. The results allowed us to (i) track the evolutionary relationships inside the genus, (ii) describe two new karyotypes, and (iii) to describe the relationship of *H. aimara* and the *H. lacerdae* group. More studies are required to clarify the relationship of the giant and common trahiras.

## Figures and Tables

**Table 1:** Species, sampling localities, and the number of specimens analyzed with cytogenetics. (PA, SC, MT, MG, and BA = the Pará, Santa Catarina, Mato Grosso, Minas Gerais, and Bahia Brazilian states, respectively).

Species	Locality	N
*Hoplias australis*	Jacutinga river, Arabutã—SC (27°10′11.1″ S 52°05′08.1″ W)	07♀, 04♂
*H. curupira*	Parauapebas river, Canaã dos Carajás—PA (6°30′06.5″ S 50°02′35.3″ W)	05♀, 03♂
*H. aimara*	Xingu river, Querência—MT (12°35’10.1″ S 52°56’35.6″ W)	01♀, 03♂
*H. intermedius*	Cipó river, Santana de Pirapama—MG (18°53’52.5″ S 43°52’58.7″ W)	04♀, 04♂
*H. brasiliensis*	Paraguaçu river, Itaberaba—BA (12°44’56.8″ S 40°12’16.0″ W)	02♀, 08♂
*H. lacerdae*	Juquiá river, Registro—SP (24°24’01.6″ S 47°49’46.8″ W)	06♀, 02♂

**Table 2:** Comparative genetic diversity levels among *Hoplias aimara* and *Hoplias lacerdae* species groups. N, number of samples; *A*_R_, allelic richness; *P*_A_, private alleles; *H*_O_, observed heterozygosity; *H*_E_, expected heterozygosity; *S*, Shannon diversity.

Species	N	*A*_R_	*P*_A_	*H*_O_	*H*_E_	*S*
*H. curupira*	3	1.006	652	0.003	0.002	1.004
*H. australis*	6	1.048	270	0.016	0.014	1.030
*H. aimara*	3	1.005	1512	0.003	0.002	1.004
*H. intermedius*	6	1.024	64	0.009	0.009	1.018
*H. brasiliensis*	6	1.049	130	0.017	0.015	1.031
*H. lacerdae*	5	1.011	184	0.005	0.004	1.008

## Data Availability

DarTSeq genotypes: dryad doi to be deposited upon acceptance.
